# Development of nematode resistance in Arabidopsis by HD-RNAi-mediated silencing of the effector gene *Mi-msp2*

**DOI:** 10.1038/s41598-019-53485-8

**Published:** 2019-11-22

**Authors:** Ila Joshi, Anil Kumar, Ashish K. Singh, Deshika Kohli, K. V. Raman, Anil Sirohi, Ashok Chaudhury, Pradeep K. Jain

**Affiliations:** 1 ICAR-National Institute for Plant Biotechnology, PUSA Campus, New Delhi, 110012 India; 2 Department of Bio & Nano Technology, Guru Jambheshwar University of Science and Technology, Hisar, 125001 Haryana India; 3 Division of Nematology, ICAR-Indian Agricultural Research Institute, New Delhi, 110012 India

**Keywords:** Molecular engineering in plants, RNAi

## Abstract

Root-knot nematodes (RKNs) are devastating parasites that infect thousands of plants. As RKN infection is facilitated by oesophageal gland effector genes, one such effector gene, *Mi-msp2*, was selected for a detailed characterization. Based on domain analysis, the Mi-MSP2 protein contains an ShKT domain, which is likely involved in blocking K^+^ channels and may help in evading the plant defence response. Expression of the *Mi-msp2* gene was higher in juveniles (parasitic stage of RKNs) than in eggs and adults. Stable homozygous transgenic *Arabidopsis* lines expressing *Mi-msp2* dsRNA were generated, and the numbers of galls, females and egg masses were reduced by 52–54%, 60–66% and 84–95%, respectively, in two independent RNAi lines compared with control plants. Furthermore, expression analysis revealed a significant reduction in *Mi-msp2* mRNA abundance (up to 88%) in female nematodes feeding on transgenic plants expressing dsRNA, and northern blot analysis confirmed expression of the *Mi-msp2* siRNA in the transgenic plants. Interestingly, a significant reduction in the reproduction factor was observed (nearly 40-fold). These data suggest that the *Mi-msp2* gene can be used as a potential target for RKN management in crops of economic importance.

## Introduction

Attacking more than 2000 plant species and leading to crop losses of 173 billion USD annually in worldwide agriculture, root-knot nematodes (RKNs; *Meloidogyne* spp.) are among the most injurious plant parasitic nematodes (PPNs)^1^. With the challenges of the ever-increasing global population and meeting food requirements, it has become important to control the damage to various crops caused by nematodes. However, the above-ground symptoms of RKN infections are not conspicuous, and these sedentary endoparasites are thus mostly unnoticed, resulting in large-scale damage. Due to their wide host range, their dynamic distribution and the associated large economic losses, RKNs are considered to be among the most threatening PPN pests. RKN second-stage juveniles (J2s) infect plant roots and form multinucleated polyploid feeding cells, termed giant cells, which are required for nematode development and reproduction^2^.

The development of specialized structures in nematodes that enable parasitism has been observed during evolution^3^. These specialized structures, such as the stylet, amphids and oesophageal glands, are required for penetration and feeding to promote parasitism^4,5^. In PPNs, parasitism is enabled by the secretion of effector proteins that suppress the host defence system. Effector proteins that are produced in a granulated form in the oesophageal glands (secretory proteins) are transferred to plant cells via the stylet^6^. These proteins are essential for the onset and maintenance of parasitism and are mainly associated with three functions: (i) facilitation of migration, (ii) defence against plant responses and (iii) establishment and maintenance of permanent feeding sites through manipulation of the host cellular machinery^7^. There are two types of oesophageal secretory glands in RKNs: subventral glands, which are responsible for the early onset of parasitism; and a single dorsal gland, which is active in later stages for maintenance of parasitism. Hence, significant changes in secreted and associated components occur during the nematode parasitic life cycle^4,8,9^. Indeed, maintenance of successful parasitism depends on interactions and molecular communication between pathogen effector proteins and host metabolic processes. Various effector genes have been reported to be involved in modulating host signalling pathways, providing evidence that endoparasitic nematodes hijack host cellular machinery to expand feeding sites. Additionally, host defence responses have been shown to be effectively altered by nematodes^10^. The roles of several effector genes in PPNs have been demonstrated; for instance, effectors such as 16D10 reportedly increase host plant vulnerability after nematode infection^11^. Additionally, overexpression of the effector protein Mi-MSP18 in onion cells led to increased susceptibility to *M*. *incognita*, *M*. *javanica* and *M*. *graminicola* infection^12^. The calreticulin effector gene of *M*. *incognita* has been found to suppress basal immunity within the host plant^13^, and another effector gene, *Mj*-FAR-1, has been reported to affect various defence-related plant responses, including on cell wall-related genes^14^. Another nuclear-localized effector protein, *Mj*-NULG1, is reportedly localized to the plant host nucleus and to possibly be involved in cell cycle modifications, and 8D05 is involved in water transport by interacting with the host protein TIP2^15,16^.

With the discovery of gene expression control via small interfering RNA (siRNA) and microRNA (miRNA) molecules, biologists have been exploring genes and development from a new perspective. For example, ingestion of double-stranded RNA (dsRNA) by *C*. *elegans*, and subsequently cyst nematodes, was reportedly able to induce posttranscriptional silencing of the complementary gene in the nematode^17,18^. Methods for *in vivo* dsRNA delivery have proven to be useful in functional analyses of nematode parasitism genes, providing a feasible gene silencing approach for investigating nematode effector function, especially for parasitism genes that are expressed only when nematodes are present in the host plant. In addition, host-delivered RNAi (HD-RNAi)-mediated resistance has been employed to target secretory proteins such as *8D05* in *Arabidopsis* and *16D10* in grape hairy roots^19,20^. Other specific genes encoding putative *M*. *incognita* oesophageal gland cell secretory proteins (*msp*s) have also been successfully silenced via HD-RNAi, including *Misp12*, *Mimsp40*, *Mimsp18*, *Mimsp20 and Mi-msp-1*^21–25^. Possible future applications of functional genomics may include the control of PPNs by validation of these putative target genes and their utilization in a loss-of-phenotype strategy through a reverse-genetic approach. In general, characterization of the effector proteins that a nematode secretes into its host during infection using the HD-RNAi technique is necessary for developing an effective way to control this devastating pest.

Previous studies on effector genes have led to the conclusion that these genes comprise good targets for HD-RNAi-mediated silencing in terms of the reduced infection rates and gene expression levels that are achieved. Secretory gland genes are crucial for the secretion of several effector molecules that have been implicated in nematode parasitism. To date, 37 putative effectors have been identified and localized subcellularly as proteins that are secreted by the subventral and dorsal oesophageal glands in *M*. *incognita*^26^.

As characterization of these effectors will lead to improved understanding of the molecular basis of *M*. *incognita* parasitism, in this study, we characterized one such *M*. *incognita* effector gene, *Mi-msp2* (for **M**eloidogyne **s**ecretory **p**rotein), as previously reported by Huang *et al*.^26^. The protein encoded by the *Mi-msp2* gene has been designated 2G02^26^, and herein we refer to this gene as *Mi-msp2*. Using HD-RNAi technology, transgenic *Arabidopsis thaliana* lines expressing *Mi-msp2* dsRNA were employed to promote nematode resistance.

## Results

### *In silico* analyses of the *Mi-msp2* gene and protein sequence

**Figure 1:**
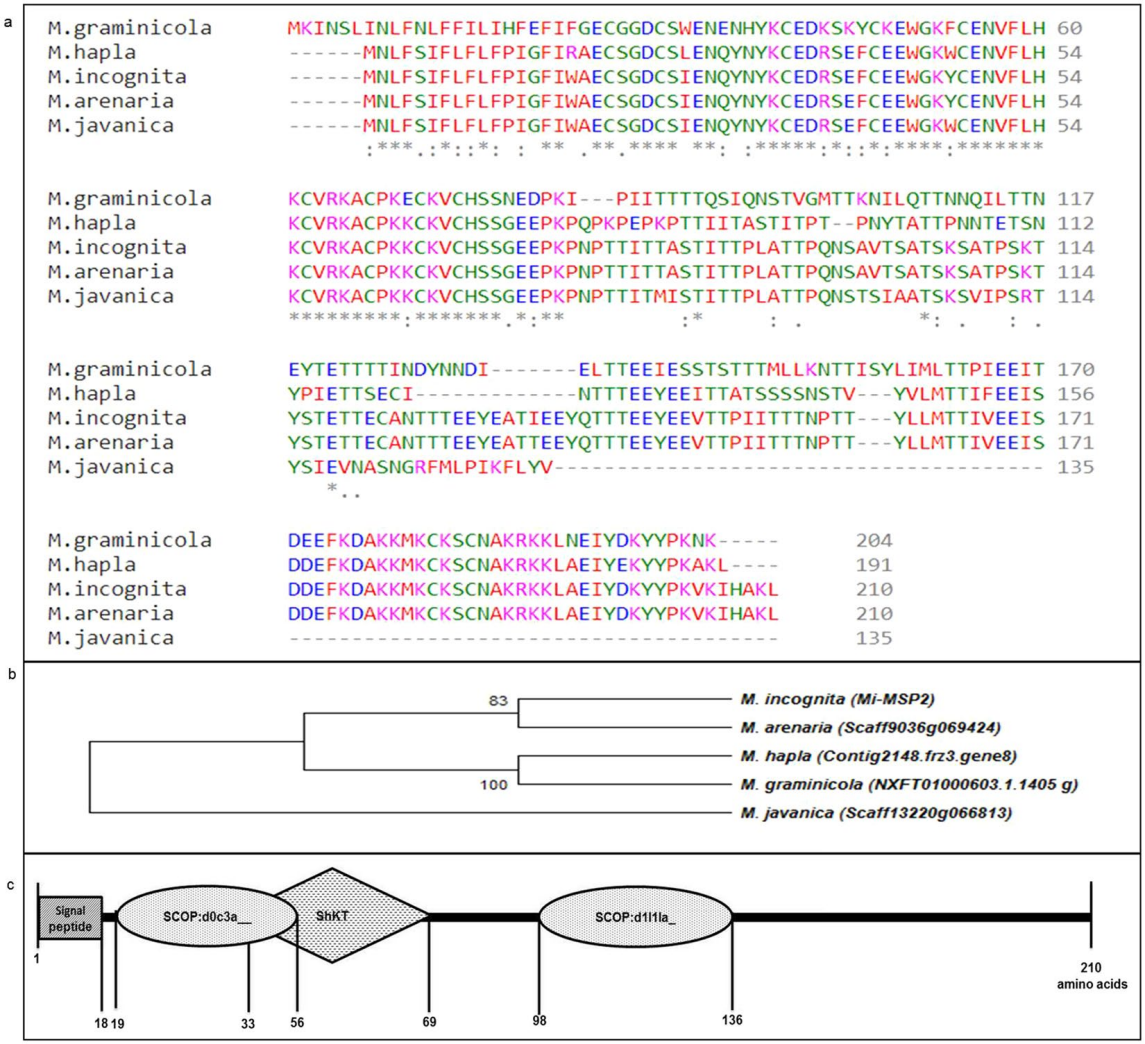
*In silico* analyses of *Mi*-MSP2 protein sequences. (**a**) Multiple sequence alignment of predicted orthologues of the *Mi*-MSP2 protein across different plant parasitic nematodes. An asterisk (*) indicates a position of a single, fully conserved residue, a colon (:) indicates conservation between groups of strongly similar properties, and a period (.) indicates conservation between groups of weakly similar properties. (**b**) The neighbour-joining phylogenetic tree was constructed using MEGA7 of *Mi*-MSP2. The percentage of replicate trees in which the associated taxa clustered together in the bootstrap test (2000 replicates) is shown next to the branches. (**c**) Illustration of the *Mi*-MSP2 protein structure and domains predicted by SMART software.

A genome-wide NCBI-BLAST analysis of the *Mi-msp2* gene sequence revealed it to be specific to *M*. *incognita*, with no sequence orthologues found in the NCBI redundant database. However, WormBase analyses led to the identification of Mi-MSP2 orthologues in three RKN *Meloidogyne* species, namely, *M*. *hapla* (Contig2148.frz3.gene8), *M*. *arenaria* (Scaff9036g069424) and *M*. *graminicola* (NXFT01000603.1.1405_g), with up to 46% of the amino acids being identical (97 of 210 amino acids) and 18% being similar (38 of 210 amino acids). Furthermore, nearly 86% of the residues towards the amino-terminal end of the protein are identical or similar, indicating extensive sequence conservation (Fig. 1a).

Structural analyses of the Mi-MSP2 protein revealed the presence of two structural classifications of proteins (SCOP) domains, SCOP:d0c3a and SCOP:d1|1|a, and one ShK toxin domain comprising 37 amino acids (residues 33–69) (Fig. 1c). The signal peptide sequence is predicted by both Signal 4.0 and SMART software to correspond to amino acids 1–18. Transmembrane domains are absent in the Mi-MSP2 protein, as depicted by TMHMM server v. 2.0 analysis, suggesting the secretory nature of the protein.

### Upregulation of the *Mi-msp2* gene in the early parasitic stage of *M*. *incognita*

**Figure 2:**
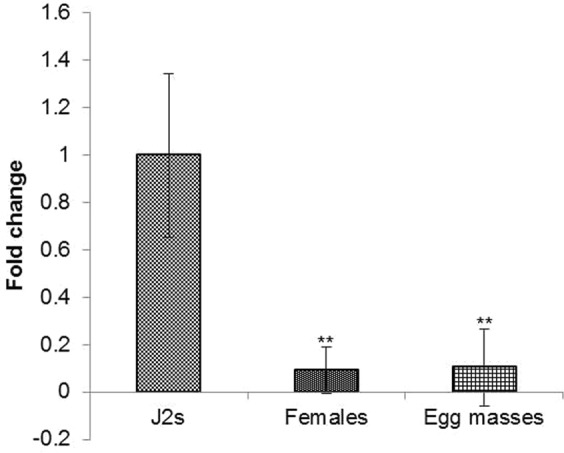
Expression analysis of the *Mi-msp2* gene at different developmental stages in *Meloidogyne incognita*. Error bars represent ± SE among the biological replicates. Double asterisks indicate statistically significant differences according to one-way ANOVA and Tukey’s test (p ≤ 0.01).

Quantitative real-time PCR analysis was performed to quantify the expression levels of the *Mi-msp2* secretory gene in three different developmental stages during the life cycle of *M*. *incognita*, namely, egg masses, J2s and adult females, isolated from RKN-infected tomato (Pusa Ruby) plants. The highest level of *Mi-msp2* expression was observed in J2s, the parasitic stage, followed by egg masses and, finally, adult females (Fig. 2). The differential levels of expression at different developmental stages illustrate the functional pattern of the secretory gland gene and its origin. The maximum expression levels found in early parasitic stage (J2s) and the subsequent decline in the adult stage suggest that *Mi-msp2* plays an important role during the early stages of parasitism.

### Infection assay of transgenic *Arabidopsis thaliana* plants expressing *Mi-msp2*

**Figure 3:**
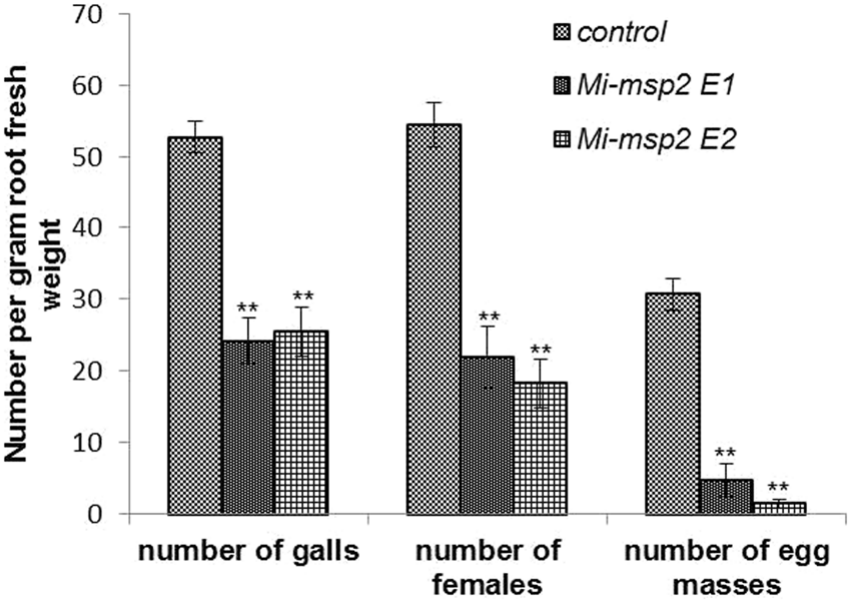
Two independent RNAi lines (*Mi-msp2* E1 and *Mi-msp2* E2) showing reductions in the numbers of galls, females and egg masses relative to control plants in response to *M*. *incognita* infection. Each bar represents the mean ± SE. Double asterisks indicate statistically significant differences according to one-way ANOVA and Tukey’s test (p ≤ 0.01).

**Figure 4:**
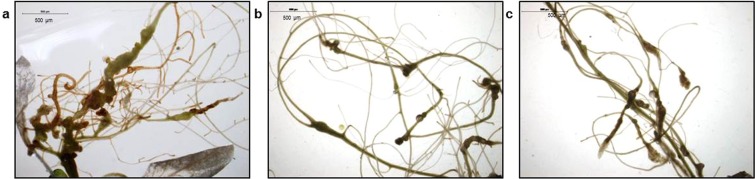
Representative images showing *M*. *incognita* in infected roots. (**a**) Roots of a control plant, (**b**) an *Mi-msp2* E1 plant, and (**c**) an *Mi-msp2* E2 plant.

A nematode infection assay was carried out using two independent homozygous T_3_ transgenic RNAi lines (*Mi-msp2* E1 and *Mi-msp2* E2) expressing *Mi-msp2* dsRNA; the number of galls, females and egg masses per gram root fresh weight were compared with those of control *Arabidopsis* plants (Fig. 3). A decrease in infection was clearly evident in the roots of both independent RNAi lines compared to the control plants (Fig. 4). The reductions in the number of galls, females and egg masses were found to be in the ranges of 52–54%, 60–66% and 84–95%, respectively. Thus, a significant reduction in nematode parasitism was observed with *Mi-msp2* gene RNAi, indicating an important role of this gene in nematode development.

### Comparative morphology of nematode females feeding on control and transgenic *Arabidopsis*

**Figure 5:**
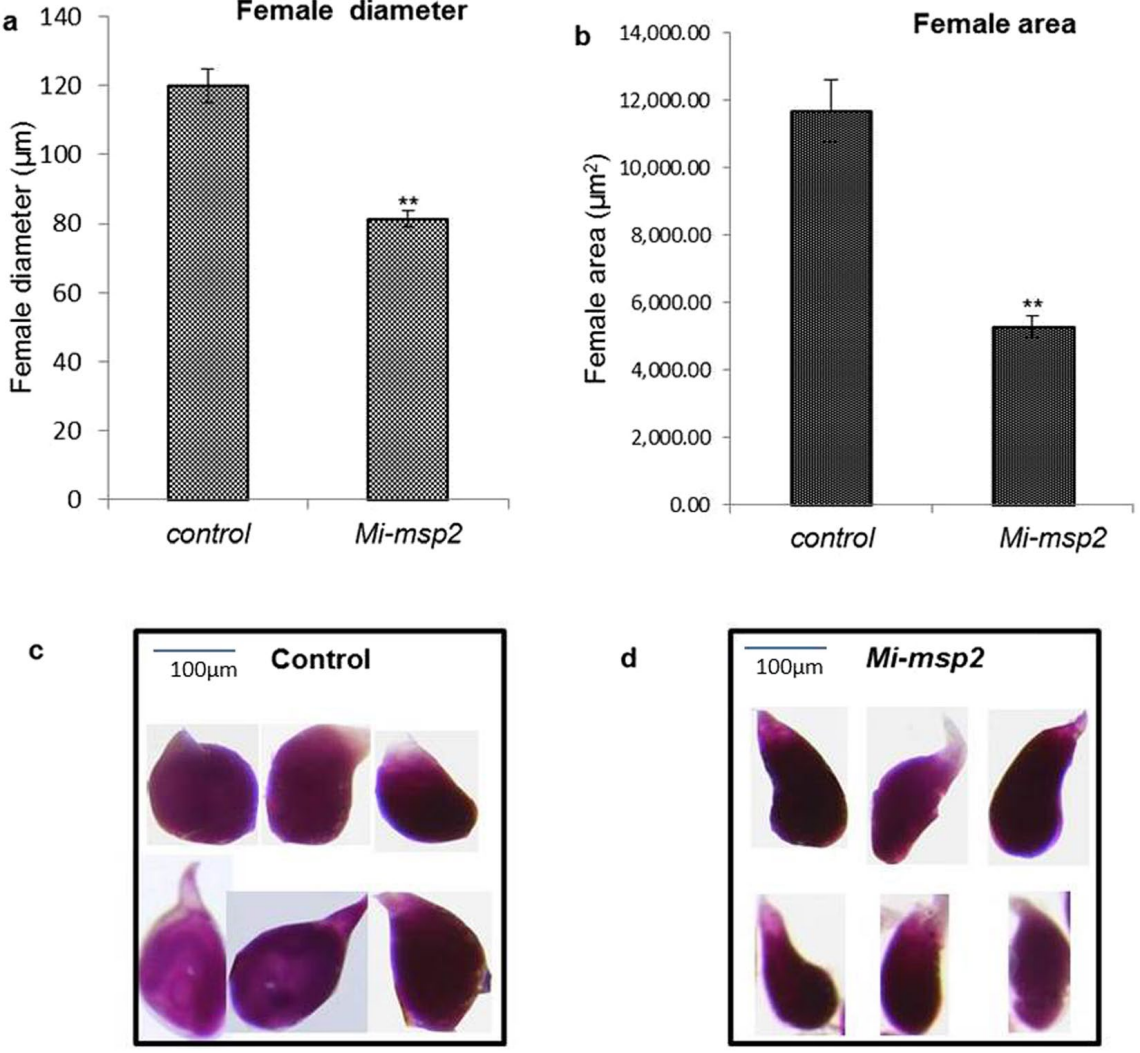
Morphological size comparison of female nematodes isolated from control and transgenic plants (cumulative females from both the *Mi-msp2* E1 and *Mi-msp2* E2 lines). (**a**) Average female width (μm), (**b**) average female area (μm^2^). Each bar represents the mean ± SE. Double asterisks indicate statistically significant differences according to one-way ANOVA and Tukey’s test (p ≤ 0.01). Representative images of adult females isolated from (**c**) control and (d) *Mi-msp2-*RNAi lines.

A comparative analysis of the size of adult female nematodes feeding on the control (wild-type) and transgenic lines was performed by measuring the width (μm) (Fig. 5a) and surface area (μm^2^) of feeding females (Fig. 5b). Twenty-five females were collected from control or transgenic plants (cumulative females from the two independent lines, *Mi-msp2* E1 and *Mi-msp2* E2), and their sizes were measured. In comparison with the control, the average width and area of females feeding on the RNAi lines were significantly reduced, with decreases in the average width and area by 32.2% and 54.8%, respectively (Fig. 5c,d), highlighting the importance of the *Mi-msp2* gene in the normal development of RKN.

### Fecundity of *M*. *incognita* females feeding on RNAi lines and control plants

**Figure 6:**
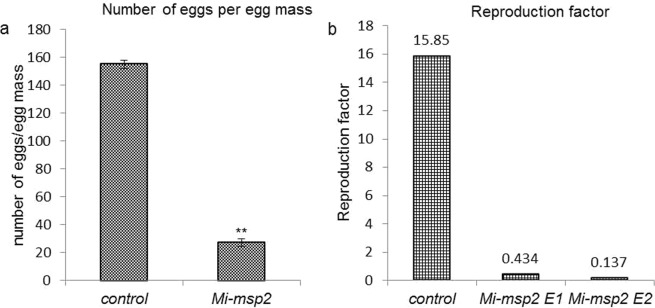
Fecundity of *M*. *incognita*. (**a**) The average number of eggs per egg mass isolated from control and transgenic plants (cumulative egg masses from both *Mi-msp2* E1 and *Mi-msp2* E2 lines). Each bar represents the mean ± SE. Double asterisks indicate statistically significant differences according to one-way ANOVA and Tukey’s test (p ≤ 0.01), (**b**) Reproduction factors for the control, *Mi-msp2* E1 and *Mi-msp2* E2.

To examine the level of susceptibility of *M*. *incognita* to HD-RNAi, egg masses isolated from control and transgenic plants (cumulative egg masses from the two independent lines, *Mi-msp2* E1 and *Mi-msp2* E2) were counted, and the number of eggs was reduced by 74.3% in the case of the *Mi-msp2* RNAi lines (Fig. 6a). Compared to that of nematodes that developed from the females feeding on control plants, the reduction in the number of eggs indicated a stalling of nematode development due to the relative inactivity of the secretory gene. In addition, the reproduction factor was calculated by measuring the number of eggs that hatched in the next generation, with values for *Mi-msp2* E1 and *Mi-msp2* E2 of 0.43 and 0.137, respectively, compared to 15.85 for control plants (Fig. 6b).

### Gene expression analysis of nematode females feeding on RNAi lines expressing dsRNA and northern hybridization analysis

**Figure 7:**
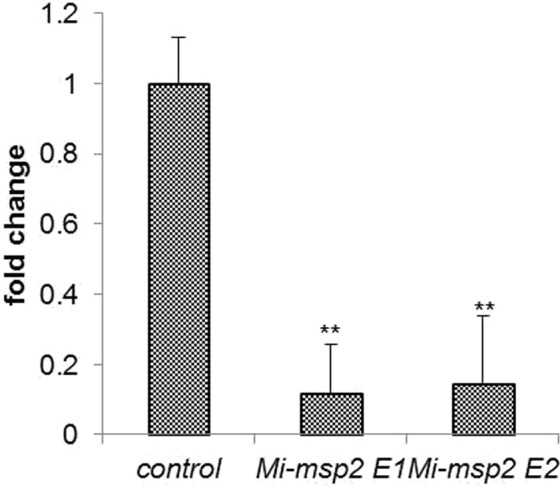
qRT-PCR-based *Mi-msp2* expression analysis of *M*. *incognita* females isolated from infected roots of control plants and two independent RNAi lines, *Mi-msp2* E1 and *Mi-msp2* E2. Each bar represents the mean ± SE. Double asterisks indicate statistically significant differences according to one-way ANOVA and Tukey’s test (p ≤ 0.01).

**Figure 8:**
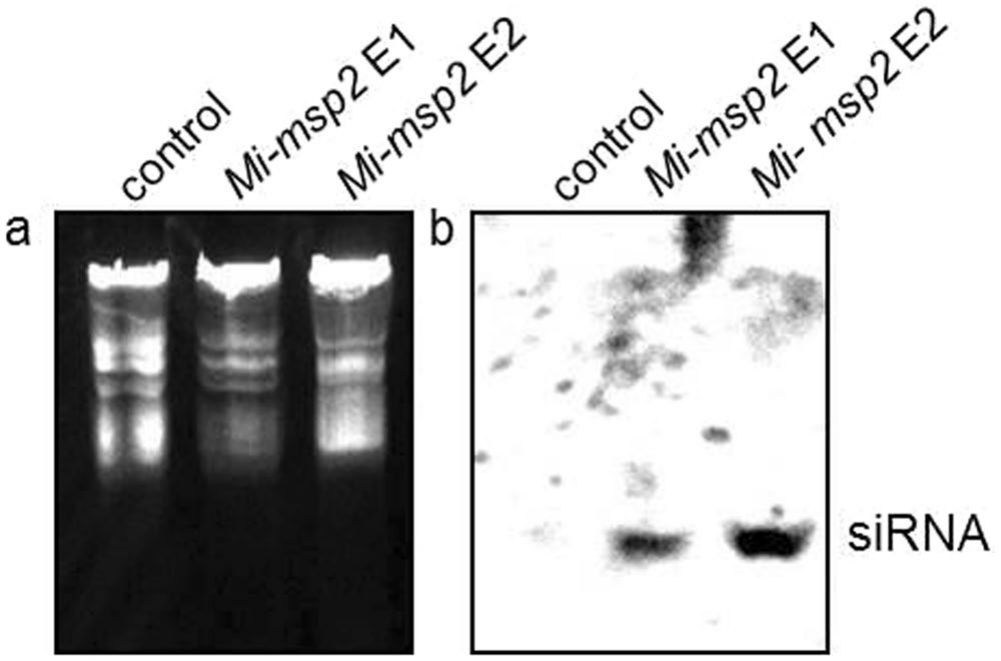
PAGE gel image. (**a**) Ethidium bromide-stained PAGE gel (before transfer) showing the total RNA fraction and (**b**) the corresponding northern blot for detecting the presence of *Mi-msp2* siRNA in control plants and RNAi lines. The full-length gel and blot images are provided in Supplementary Fig. S3.

qRT-PCR was performed to examine *Mi-msp2* gene expression in females isolated from transgenic plants compared to control plants, and a reduction in gene expression was observed in terms of the fold change calculated using ΔΔCt values, as evaluated against nematode *actin*^27^. The decrease in gene expression was 0.882-fold for *Mi-msp2* E1 and 0.855-fold for *Mi-msp2* E2 (Fig. 7) in comparison to the control. siRNA specific for *Mi-msp2* was also detected in the RNAi lines by northern hybridization (Fig. 8), further confirming successful RNAi in these lines. Thus, the decreased infection rate may be attributed to silencing of the *Mi-msp2* gene, highlighting the ability to use HD-RNAi to promote resistance as an important strategy for managing RKNs.

## Discussion

PPNs are a consistent threat to agricultural crops. However, the increasing amount of information regarding PPN genomes and mechanism(s) of parasitism that has become available in the past 15 years has enabled new strategies for combating one of the most threatening crop parasites. Indeed, the advent of RNAi technology has provided a very promising tool for combatting these menacing nematodes^28^. In addition, with the aim of conferring nematode resistance, genome mining has led to the characterization and isolation of various nematode genes and promoters involved in successful parasitism. Various nematode genes involved in parasitism have been identified and employed using an HD-RNAi-mediated approach to develop nematode-resistant plant^8,13,29,30^. In addition to effector genes, other nematode genes that have been targeted include housekeeping genes, such as a splicing factor and integrase, transcription factors, ribosomal protein 3a, ribosomal protein 4, and developmental genes encoding spliceosomal SR proteins, such as *glp-1* and *col1*, a dual oxidase gene^31–37^. Although targeting these genes has reduced the level of PPN parasitism, greater and more effective reductions in infection have been achieved by silencing effector genes. The genes targeted include effector genes such as *16D10*, *8D05*, *Mimsp40*, *Hs4G06*, *Hs3B05*, *Hs8H07*, and *Hs10A06* and those encoding cell wall-degrading enzymes such as cellulose-binding proteins and β-1,4-endoglucanase^11,19,22,27,38,39^. Interesting and encouraging results have been obtained by targeting the *16D10* and *Hg30C02* genes, with up to a 90% reduction in nematode infection observed^11,40^.

The present study involved the utilization of *Mi-msp2* (AF531161), an effector gene, as a potent target for an HD-RNAi-mediated approach to manage nematode parasitism. *Mi-msp2* was previously reported by Huang *et al*. to be an effector protein of subventral gland origin in *M*. *incognita*^26^. BLAST analysis using WormBase led to the identification of orthologues for the *Mi*-MSP2 protein in all four *Meloidogyne* species. However, this result was in contrast to that of a similar analysis using the NCBI database, which identified no orthologues. The Mi-MSP2 protein sequence shows extensive conservation with the MSP2 proteins of other *Meloidogyne* species.

A detailed computational analysis of the *Mi-msp2* gene structure, as well as the protein structure, was performed. The absence of a transmembrane subunit revealed the secretory nature of the Mi-MSP2 protein, similar to other secretory proteins; these proteins are directly secreted from the nucleus into the oesophageal gland cell lumen in the form of membrane-bound granules derived from the Golgi apparatus^3^. Protein domain structure predictions for the Mi-MSP2 protein carried out using SMART software revealed the presence of two SCOP domains, SCOP:d0c3a and SCOP:d1|1|a. The SCOP:d0c3a domain belongs to the C3a anaphylatoxin family of the complement system; the SCOP:d1|1|a_domain belongs to the pyruvate formate-lyase-like (PFL) glycyl radical superfamily, which is involved in oxidation-reduction reactions in several metabolic processes. We were unable to find any previous reports of the existence of the C3a complement domain in nematodes. Another domain present in the Mi-MSP2 protein is the *Stichodactyla* toxin (ShKT; from the sea anemone *Stichodactyla helianthus*) domain, which has an ancient origin in both plants and animals. The function of this domain in worms (*Caenorhabditis elegans*, *Brugia malayi*, *Ancyclostoma ceylanicum*, *Schistosoma mansoni*) is specifically related to the modulation of ion channels^41^. In *C*. *elegans*, the ShKT domain is found in the astacin/adamalysin family of metallopeptidases, which function in blocking voltage-gated K^+^ channels (Kv). The ShK toxin is heat resistant and binds to the phospholipase A subunit of K^+^ channels, blocking the influx of K^+^. As K^+^ channels are reported to be indirectly involved in plant defence responses through the action of reactive oxygen species (ROS) (biotic and abiotic stress responses)^41^, it is plausible that the ShK toxin has a similar effect on Kv channels in plants. Two 37-aa putative C-terminal ShKt domains are also present in another sub-ventral gland gene, *Mi msp40*, of *M*. *incognita*, which suggests that the functionality of these domains in host infection is mediated by blocking Kv channels^22^. In the case of animal-parasitic worms, ShK toxin has been found to exhibit an immunomodulatory effect, and this toxin has been employed as a selective inhibitor of Kv channels to treat autoimmune disease^42^.

Huang *et al*. reported the subcellular localization of putative effector proteins in *M*. *incognita*^26^. Consistent with their findings, our study on stage-specific gene transcription revealed that *Mi-msp2* (subventral oesophageal gland origin) was expressed at higher levels during the initial parasitic developmental stages than in the other stages investigated, with maximal expression was observed in J2s and egg masses^43,44^.

HD-RNAi-mediated silencing of *Mi-msp2* resulted in a reduction in the number of galls (up to 54%) in transgenic *Arabidopsis* RNAi lines compared to the control, illustrating the importance of the *Mi-msp2* secretory gene for successful parasitism. Similar results regarding a reduction in the number of galls were observed for two housekeeping genes encoding secretory splicing and integrase proteins, *16D10* (63–90% reduction) and *8D05*^11,19,31^. In our analyses, the number of gall-inhabiting females that were able to successfully parasitize plants was reduced by 60–66%. Completion of the nematode life cycle is marked by the egg masses laid by adult females, and the number of egg masses was drastically reduced by 84–95% in the RNAi lines. This finding provides useful insight into the mode of *M*. *incognita* parasitism and the relevance of secretory genes in host invasion. Furthermore, comparison of the reductions in the numbers of females and egg masses revealed a dramatic decrease in the number of egg masses, which indicates the importance of secretory genes in reproduction and fecundity. The results reported by Kumar *et al*. regarding RNAi targeting of splicing and integrase genes showed the same pattern of a decreased number of egg masses compared to the number of females^32^. The reduction in fecundity was further confirmed by analysis of the number of eggs per egg mass in the RNAi lines, demonstrating a strong decline in the number of eggs by up to 74%, similar to the decrease reported for *16D10*^11^.

As *Mi-msp2* expression was reduced by up to 88% in the transgenic RNAi lines, we can infer its importance in *M*. *incognita* parasitism. Further studies on the protein function of Mi-MSP2 should be performed to assess its relevance regarding parasitism and development. Moreover, comparison of female size (in terms of width and area) indicated that along with hampering parasitism, RNAi knockdown of this effector gene impeded development. This observation further demonstrates the relevance of investigating secretory gene function Additionally, gene expression analyses conducted to elucidate the role of *Mi-msp2* in the normal progression of nematode parasitism revealed a reduction in transcript levels in the RNAi lines. Thus, the reduction in infection can be linked to the reduction in transcript levels, suggesting a lack of functional redundancy. In support of this observation, the detection of siRNA in the RNAi lines confirmed that the decrease in nematode infection was due to successful HD-RNAi-mediated knockdown of the *Mi-msp2* gene. Thus, HD-RNAi knockdown of the *Mi-msp2* gene may be considered successful, as demonstrated by a reduction in the levels of gene expression. Based on our results, *Mi-msp2* appears to be an important candidate gene, reducing nematode infection by as much as 88%, and may be employed for promoting nematode resistance in economically useful plants. Furthermore, because of the significant sequence conservation, the gene may be applied for the management of different RKNs (*Meloidogyne* spp.).

Several candidate genes have been identified, many of which have exhibited the ability to reduce nematode infection, with varying efficiencies, after being targeted by the HD-RNAi-mediated approach. This result is truly encouraging, further identification and extensive characterization of gene(s) in different plant/crop systems should be performed to obtain and utilize the best combination for addressing nematode infection. Undoubtedly, effector genes are the most important targets in the arsenal against nematode infection, and their utilization should be a successful approach to managing PPNs.

## Materials and Methods

### Gene and protein sequence analysis of *Mi-msp2*

The 590-base pair sequence of *Mi-msp2* (AF531161), reported by Huang *et al*., was subjected to BLAST analysis across the NCBI redundant database^11^ as well as a WormBase BLAST/BLAT search (https://wormbase.org/tools/blast_blat). Multiple sequence alignment was performed for the orthologues of the Mi-MSP2 protein across *Meloidogyne* spp. using CLUSTAL Omega software; and simple phylogeny analysis was achieved with MEGA-X software^45,46^. Domain analysis of the Mi-MSP2 protein sequence was performed using the SMART database to search for different structural domains^47^. The signal peptide sequence was identified using SignalP3.0, and the TMHMM server v. 2.0 was employed to predict the presence of transmembrane structures^48,49^.

### Preparation of *M*. *incognita* effector gene RNAi constructs

The *Mi-msp2* dsRNA construct was prepared using Gateway technology. For bacterial recombination, *attB1* and *attB2* sites were added to gene-specific primers. PCR amplification of the *Mi-msp2* effector gene (590 bp) was conducted using *M*. *incognita* cDNA, and the product was cloned into the pGEM-T Easy vector (Promega, USA). Subsequently, the size and sequence of the fragment were confirmed through Sanger DNA sequencing, and the fragment was subcloned into the pDONR vector pRK2031 using BP clonase (Invitrogen, USA)^50^. Positive clones were then transferred into a plant expression vector, pYSB (this vector was generated as part of the current study in collaboration with IIT Kanpur, and it drives expression of sense and antisense genes using the 35S promoter), using the Gateway LR clonase II enzyme mix (Invitrogen, USA) to obtain the recombinant expression vector. The recombinant pYSB RNAi (Fig. S1) constructs were transferred into *Agrobacterium tumefaciens* strain GV3101 by the freeze-thaw method according to the standard procedure^51^. For the selection of recombinant transformants, *Agrobacterium* was grown on YEP medium containing 50 mg/L hygromycin and 25 mg/L rifampicin. Colony PCR was performed to confirm positive clones, with introduction into *Arabidopsis thaliana* via *Agrobacterium-*mediated transformation^52^. The constructs were screened using MS medium containing 50 mg/L kanamycin. PCR confirmed transgenic RNAi plants expressing the *Mi-msp2* dsRNA constructs (Fig. S2), followed by propagation to the T3 homozygous generation.

### Obtaining a pure *M*. *incognita* culture and maintenance of the culture in the tomato cultivar Pusa Ruby

A pure culture of the RKN *M*. *incognita* (Kofoid & White) Chitwood race 1 was obtained from the Division of Nematology, Indian Agricultural Research Institute (IARI), New Delhi and maintained in Pusa Ruby tomato (*Solanum lycopersicum L*.) plants. Females that developed from the pure culture obtained from a single egg mass were isolated to identify *M*. *incognita* using sequence-characterized amplified region (SCAR) primers and the perineal pattern of the female^53,54^. J2s were hatched from the egg mass obtained from an *M*. *incognita* female and used to propagate the pure culture. Ten-day-old tomato seedlings were transferred to 6-inch-diameter pots containing a soil medium mixture of sand, soil rite and vermiculite in a 1:1:1 ratio. Hoagland solution was administered to the plants regularly, and the plants were infected with 500 *M*. *incognita* juveniles. After 6 to 7 weeks of infection, the roots were observed for gall development; the roots were observed for egg masses after completion of the *M*. *incognita* life cycle. The egg masses were then hand-picked with the help of a dissection needle; to allow for hatching for 3 days at 28 °C, the egg masses were placed on tissue paper over a wire gauge on Petri plates in 20–30 mL of sterile water following the modified Baermann funnel technique^55^. J2s were collected in the Petri plates to maintain a continuous culture of *M*. *incognita*.

### Development of transgenic *Arabidopsis thaliana* lines containing the effector gene *Mi-msp2*

*Arabidopsis thaliana* (Col-0) plants were transformed with the *Mi-msp2* RNAi construct using the floral dip method^52^. T_1_ seeds of transgenic plants expressing dsRNA for the *Mi-msp2* gene were sterilized and grown on MS medium supplemented with 1% sucrose and 0.8% agarose. The transformants were selected on growth medium supplemented with 50 µg/mL kanamycin. Seedlings that were resistant to kanamycin and produced healthy green leaves and well-established root systems were selected and transferred onto an autoclaved soilrite mixture and grown to maturity in a greenhouse at 22 °C with an 8-hour light/16-hour dark photoperiod. The seeds were harvested, and molecular analysis was carried out. T_2_ seeds were used to produce T_3_ lines; all nematode infection assays were carried out on T_3_ homozygous transgenic lines. The RNAi-transgenic plants were subjected to phenotypic comparison with control plants to exclude variables related to nematode infection. The controls and transgenic RNAi lines were observed phenotypically to assess changes in growth patterns, which can indirectly alter nematode infection. The RNAi lines were visually compared with the control on agar medium for root length and growth patterns. No gross morphological differences were observed in root growth between the RNAi lines and control plants or among the RNAi lines.

The control and RNAi plants were also assessed morphologically for any differences in terms of root and shoot growth, flowering response and life cycle, and no differences were observed. Furthermore, no phenotypic differences among the plants of different RNAi lines were found.

### Sterilization and growth of transgenic *Arabidopsis thaliana* plants

Seeds of the *A*. *thaliana* control line (wild-type Col-0) and two independent T3 transgenic lines harbouring *Mi-msp2* RNAi were sterilized with 70% ethanol (1 min) and with 0.1% SDS and mercuric chloride (8 min) and then washed three times with sterile double-distilled, autoclaved water. The sterilized seeds were vernalized on 0.1% agarose at 4 °C for 72 hours and sown on autoclaved MS medium (pH 5.8) (HiMedia). Plants were grown at 21 °C with a 16-h light/8-h dark photoperiod. Two-week-old seedlings were transferred to 9-cm-diameter round Petri dishes containing 1/2 MS and CleriGel (HiMedia), with eight plants per plate. The Petri dishes were placed at a slight angle of 45° to promote unidirectional root growth for approximately one week until secondary roots appeared.

### Sterilization of nematodes and infection assay

*M*. *incognita* J2s were hatched at 28 °C and suspended in M9 buffer before being further subjected to surface sterilization using nematode sterilization buffer, followed by several washes with autoclaved double-distilled water^56^. Three-week-old plants were infected with the sterilized *M*. *incognita* J2s (approximately 300 J2s per plant). For phenotypic analysis and collection of adult females and egg masses, the plants were carefully harvested from MS media (CleriGel) at 35 days postinoculation (dpi) and weighed to determine the fresh weight of the root mass. The numbers of galls, females and egg masses per gram of root fresh weight were counted manually by dissecting the roots and isolating the females and egg masses under light microscopy. The data obtained from the RNAi lines were used as a measure of relative infection in comparison with the control plants. The mean numbers of galls, females and egg masses per gram of root fresh weight were calculated from 10–15 biological replicates per RNAi line. The susceptibility of the control and RNAi lines was assessed by comparing the gene-silencing efficiencies.

### Calculation of the reproductive capacity of *M*. *incognita* after *Mi-msp2* gene silencing

To assess the reproductive capacity of *M*. *incognita* after gene silencing, a comparison of Oostenbrink’s reproduction factor was performed between the control and transgenic lines^57^. The reproduction factor provides a measure for assessing variation in nematode responses to host plants. The number of J2s that hatched from the next generation of egg masses was calculated. Photographs of infected roots and *M*. *incognita* females were acquired at a suitable magnification with a NIKON® microscope and associated camera software NIS element D (Nikon Instruments Inc., New York, USA). The lengths and widths of 25 nematode females were recorded and averaged using the same microscope software.

$$\begin{array}{l}\mathrm{Reproduction}\,\mathrm{factor}\,({\rm{R}})\\ =\,\mathrm{final}\,\mathrm{nematode}\,\mathrm{population}\,\mathrm{hatched}\,\mathrm{from}\,\mathrm{egg}\,\mathrm{masses}/\mathrm{initial}\,\mathrm{number}\,\mathrm{of}\,\mathrm{J2s}\,\mathrm{per}\,\mathrm{plant}\,\mathrm{for}\,\mathrm{infection}\end{array}$$

### RNA isolation and gene expression analysis of RNAi females

To study expression of the *Mi-msp2* gene, total RNA was isolated from three developmental stages of the nematode, eggs, J2s and adult females, using PureLink RNA Mini Kit (Thermo Fisher Scientific) according to the manufacturer’s protocol. RNA integrity was assessed by formaldehyde gel electrophoresis with ethidium bromide. A NanoDrop spectrophotometer 8000 (Thermo Fisher Scientific, Massachusetts, USA) was used to calculate purity ratios and quantify total RNA. The RNA (500 ng) obtained was used to prepare cDNA using SuperScript III First-Strand Synthesis System (Thermo Fisher Scientific, Massachusetts, USA), and the cDNA was used as a template for qRT-PCR (Applied Biosystems StepOnePlus™ Real-Time PCR). RT primers were designed based on the cDNA sequence of *Mi-msp2* using the online ‘Primer3’ portal (http://frodo.wi.mit.edu/primer3/) (Table S1)^58^. Gene expression analysis was performed by comparing δδCt values between females feeding on control plants and transgenic *Arabidopsis* plants expressing dsRNA targeting the secretory gene^27^. Three independent transgenic lines were evaluated using two housekeeping genes: nematode *actin* and nematode *18S* (accession numbers: Mi-actin, Minc06769; Mi-18S, P901057.2). The results obtained using the nematode *actin* gene are reported here. Statistical analysis using ANOVA and Tukey’s test was performed to assess significance.

### Northern hybridization for siRNA detection

Total RNA was isolated from the two RNAi lines and control plants using the TRIzol method. Samples of total RNA (80 μg) were resolved by 15% polyacrylamide/0.5 X TBE urea gels and then blotted onto a BrightStar™-Plus positively charged nylon membrane (15 cm × 15 cm). For probe preparation, primers for the complete sense strand were used to isolate the gene sense strand suitable for siRNA. To generate specific DNA probes (590 bp), digoxigenin labelling was performed using a DIG-RNA labelling kit (SP6/T7) (Roche). Hybridization was performed using a DIG Northern starter kit (Roche) according to the manufacturer’s instructions, which involved blocking the membrane and immunological detection with an anti-DIG-AP conjugate. Signals were detected by the chemiluminescence method using CDP Ready-To-Use solution (Applera Corporation) as the substrate.

## Supplementary information

Supplementary information
