# Supplementary material for: Development of nematode resistance in Arabidopsis by HD-RNAi-mediated silencing of the effector gene Mi-msp2
Source: Sci Rep. 2019 Nov 22;9:17404. doi: 10.1038/s41598-019-53485-8 (PMC6874571; doi:10.1038/s41598-019-53485-8)
Supplement: Supplementary file 1 — Supplementary information [file 41598_2019_53485_MOESM1_ESM.pdf]

Development of nematode resistance in Arabidopsis by HD-RNAi-mediated silencing of the effector gene *Mi-msp2*

Ila Joshi[1,2], Anil Kumar[1], Ashish K. Singh[3], Deshika Kohli[1], K. V. Raman[1], Anil Sirohi[3], Ashok Chaudhury[2] and Pradeep K. Jain[1]

[1]ICAR-National Institute for Plant Biotechnology, PUSA Campus, New Delhi 110012

[2]Department of Bio & Nano Technology, Guru Jambheshwar University of Science and Technology, Hisar 125001, Haryana, India.

[3]Division of Nematology, ICAR-Indian Agricultural Research Institute, New Delhi 110012, India

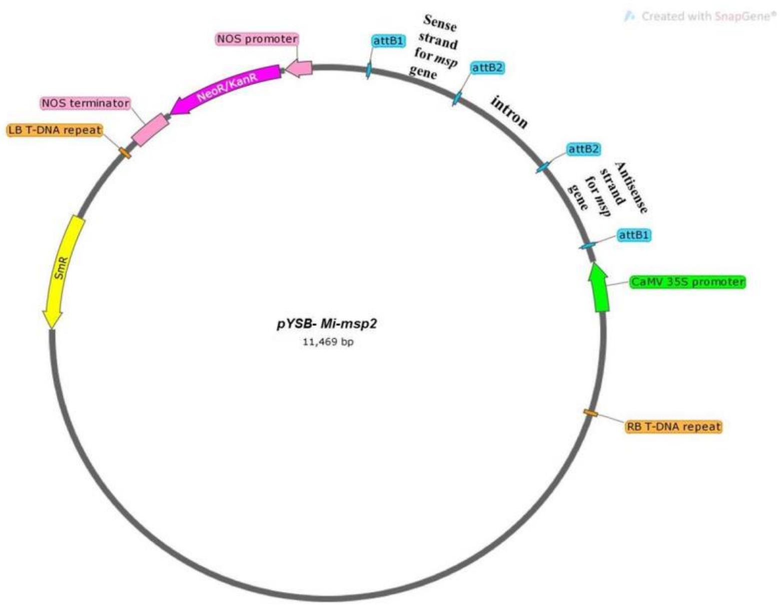

Supplementary Figure S1: Vector expressing the dsRNA construct for the *Mi-msp2* gene.

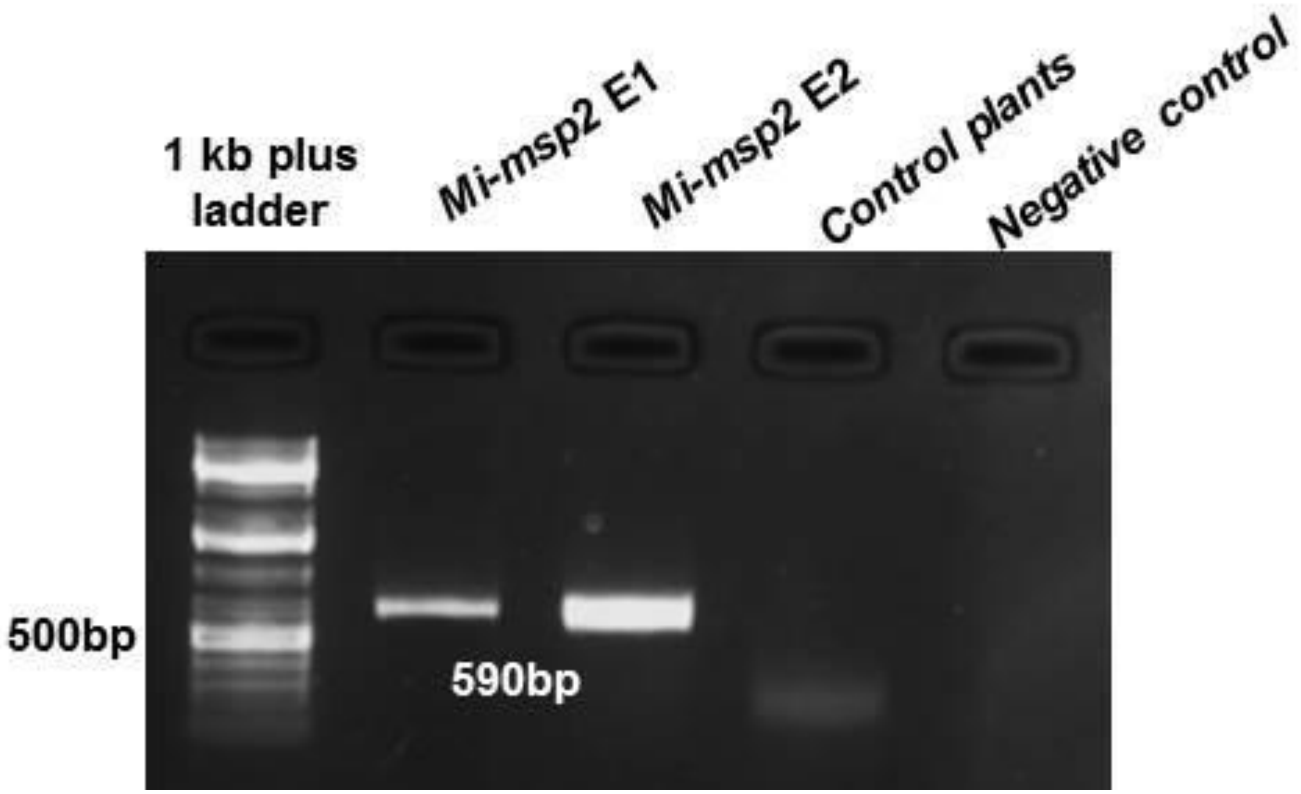

Supplementary Figure S2: confirmation of transgenic RNAi plants by PCR with *Mi-msp2* gene specific primers

a

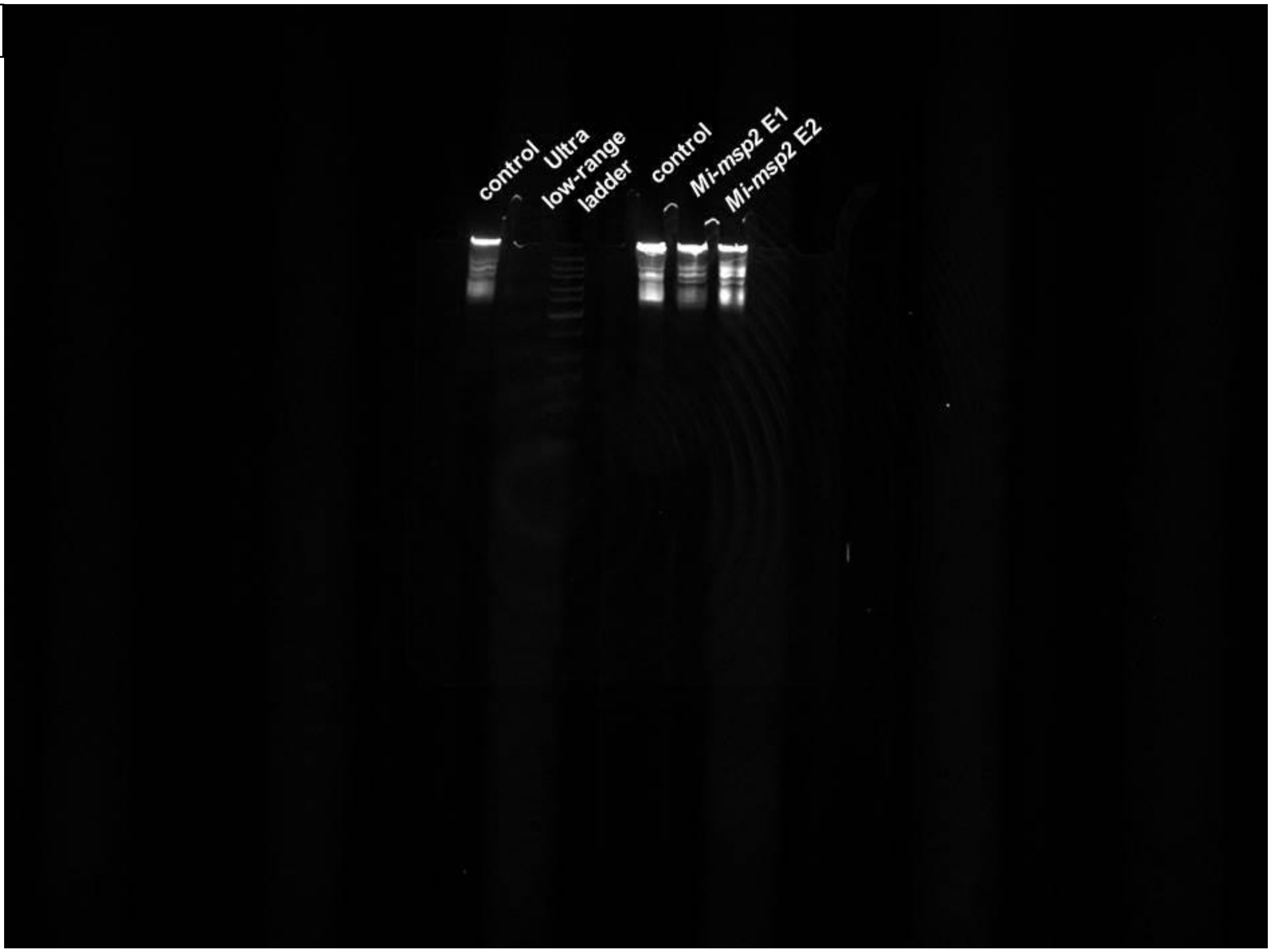

b

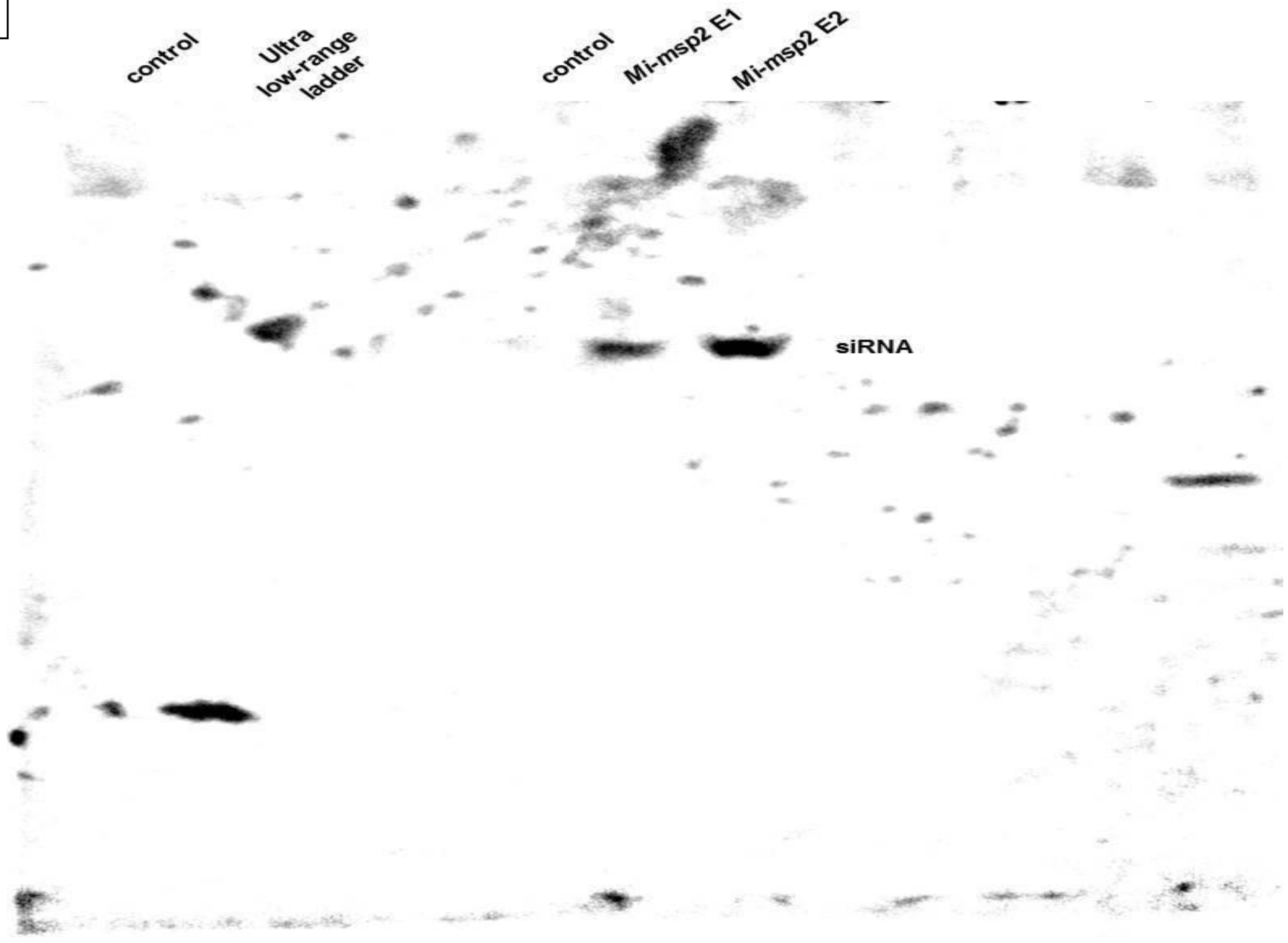

Supplementary Figure S3: Complete images for (a) PAGE gel and (b) blot for figure 8.

Supplementary Table T1: List of primers

Name of primer	Sequence (5'-3')	Product size
Primers for gateway cloning		
Mi-msp2 GW F	tctcccgggCCAATCGGGTTTATTTGGGCT	590bp
Mi-msp2 GW R	tctcccgggGCATGAATCTTAACTTTCGGA	
Primers for molecular confirmation		
Mi-msp2 gene F	CCAATCGGGTTTATTTGGGCT	590bp
Mi-msp2 gene R	GCATGAATCTTAACTTTCGGA	
Quantitative real time PCR primers		
Mi-msp2 RT 1_F	GTTACTTCGGCAACCTCAAA	237bp
Mi-msp2 RT 1_R	CTTTGCGTCTTTGAATTCGT	
Act-N1_F QTR	TACGCCAACACTGTCCTTTC	125bp
Act-N1_R QRT	CGCTCAGGAGGTGCAATAAT	
Mi 18S QRT F 1	GGCTCATGGTGGAAAGTATG	167bp
Mi 18S QRT R 1	CCCCAGTGTAATGTCCTTTG	
